# Effect of Cigarette Smoking on Epithelial to Mesenchymal Transition (EMT) in Lung Cancer

**DOI:** 10.3390/jcm5040044

**Published:** 2016-04-11

**Authors:** Trung Vu, Lin Jin, Pran K. Datta

**Affiliations:** Division of Hematology and Oncology, Department of Medicine, Comprehensive Cancer Center, University of Alabama at Birmingham, Birmingham, AL 35233, USA; ttvu@uab.edu (T.V.); jinl2@uab.edu (L.J.)

**Keywords:** epithelial to mesenchymal transition, cigarette smoke, drug resistance, metastasis, and lung cancer

## Abstract

Epithelial to mesenchymal transition (EMT) is a process that allows an epithelial cell to acquire a mesenchymal phenotype through multiple biochemical changes resulting in an increased migratory capacity. During cancer progression, EMT is found to be associated with an invasive or metastatic phenotype. In this review, we focus on the discussion of recent studies about the regulation of EMT by cigarette smoking. Various groups of active compounds found in cigarette smoke such as polycyclic aromatic hydrocarbons (PAH), nicotine-derived nitrosamine ketone (NNK), and reactive oxygen specicies (ROS) can induce EMT through different signaling pathways. The links between EMT and biological responses to cigarette smoke, such as hypoxia, inflammation, and oxidative damages, are also discussed. The effect of cigarette smoke on EMT is not only limited to cancer types directly related to smoking, such as lung cancer, but has also been found in other types of cancer. Altogether, this review emphasizes the importance of understanding molecular mechanisms of the induction of EMT by cigarette smoking and will help in identifying novel small molecules for targeting EMT induced by smoking.

## 1. Introduction of EMT

Epithelial to mesenchymal transition (EMT) is a process through which epithelial cells undergo multiple biochemical changes to acquire mesenchymal phenotype and increase migratory capacity. Based on the physiological tissue context, EMT has been categorized to take place in three types of biological processes: (1) embryonic development and organ formation [1,2]; (2) wound healing and organ fibrosis [3]; and (3) cancer progression [4]. Type 3 EMT is found to be associated with an invasive or metastatic phenotype [5]. Defining morphological changes of EMT are the dissolution of the epithelial junctions, the disruption of the polarity complex, and the reorganization of the cytoskeletal architecture. At the molecular level, EMT is characterized by the downregulation of genes encoding for epithelial cell junction proteins (E-cadherin, claudins, and occludins) and the activation of genes, the protein products of which promote mesenchymal adhesion (vimentin, fibronectin and N-cadherin) [6]. Among the expression changes of epithelial and mesenchymal markers, the downregulation of E-cadherin is considered as a hallmark of EMT that leads to destabilization of adherens junctions [7].

Upregulation of EMT-related transcription factors and mesenchymal markers has been found in lung cancer and associated with increased rate of cancer recurrence and decreased survival in lung cancer patients (Table 1). Previous studies indicated that overexpression of EMT-related transcriptional factors, such as SNAIL1 [8,9,10], SLUG [11], TWIST [12,13,14], ZEB1 [15,16], FOXC2 [17], FOXQ1 [18], FOXC1 [19], and FOXM1 [20], are associated with invasiveness, metastasis and poor prognosis of lung cancer. Lung metastatic carcinomas show a significantly higher expression of these transcription factors than primary lung tumors, indicating their importance in the metastatic process. Loss or dysfunction of E-cadherin is associated with an invasive phenotype in lung cancer [21]. Furthermore, increased expression of mesenchymal proteins, such as N-Cadherin [22], Syndecan-1 [23], miR-21 [24], Vimentin [25], Periostin [26], and alpha-smooth muscle actin (α-SMA) [27], were also found in patients with poor prognosis.

The regulated expression and activity of several EMT-associated proteins have been shown to be involved in this process. There are three major groups of EMT-activating transcription factors: The SNAIL family of zinc-finger transcription factors SNAIL1/SNAIL2, the ZEB family of transcription factors ZEB1/ZEB2, and the TWIST family of bHLH transcription factors TWIST1/TWIST2. Due to distinct expression profiles, contributions of these transcription factors to EMT depend on the cell or tissue type and the signaling pathways that initiate EMT [6].

The SNAIL family of zinc-finger transcription factors, consisting of SNAIL1, SNAIL2, and SNAIL3 (also known as SNAIL, SLUG and SMUC respectively), activate EMT program during development, fibrosis and cancer [28]. The mechanism by which SNAIL proteins repress the expression of epithelial markers is represented by the activity of SNAIL1 on the promoter of E-cadherin. SNAIL1 suppresses expression of E-cadherin by binding to E-box DNA sequences and recruiting histone deacetylases (HDACs) [29], the Polycomb repressive complex 2 (PRC2) [30,31], Lys-specific demethylase 1 (LSD1) [32], G9a [33], and suppressor of variegation 3–9 homologue 1 (SUV39H1) [34]. The assembly leads to various histone modifications including methylation and acetylation at histone H3 Lys 4 (H3K4), H3K9 and H3K27, resulting in the suppression of E-cadherin promoter activity. In addition to repressing epithelial genes, SNAIL1 activates genes that contribute to the mesenchymal phenotype, such as fibronectin, N-cadherin, and collagen. SNAIL also induces EMT through upregulating other EMT transcription factors, such as SLUG [35], TWIST, ZEB2 [36], and ZEB1 [37]. Diverse signaling pathways cooperate in the initiation and progression of EMT by activating SNAIL1 expression. SNAIL1 expression has been known to be upregulated by Notch [38], TGF-β [38], and Wnt-β-catenin [39] signaling pathways.

The family of basic helix–loop–helix (bHLH) transcription factors plays essential roles in lineage commitment and differentiation. In this family, TWIST1 and TWIST2 have a key role in EMT regulation. TWIST1 downregulates epithelial gene expression and activates mesenchymal gene expression [40] independently of SNAIL and through association with other proteins. Importantly, TWIST1 recruits the methyltransferase SET8, which repress the E-cadherin and activate N-cadherin promoter via its H4K20 mono-methylation activity [41]. TWIST expression can be upregulated by multiple signaling pathways during tumorigenesis. EGF/EGFR signaling pathways upregulate TWIST expression in a manner dependent on STAT3 [42]. Under hypoxic conditions, the expression of TWIST can be induced by the transcription factor hypoxia-inducible factor 1α (HIF1α) [43]. In addition to expression regulation, MAPKs can regulate TWIST1 by phosphorylating TWIST1 at Ser68, stabilizing it from ubiquitin-mediated degradation and increasing its activity [44].

The ZEB family of transcription factors contains two members (ZEB1 and ZEB2), which bind regulatory gene sequences at E-boxes and function as transcriptional repressors and activators, thereby repressing some epithelial genes and activating mesenchymal genes. ZEBs mainly mediate transcriptional repression by recruiting C-terminal-binding protein (CTBP) co-repressor to E-boxes [45]. In addition to CTBP, ZEB1 can also recruit Switch/sucrose non-fermentable (SWI/SNF) chromatin remodeling protein BRG1 and repress E-cadherin expression [46]. ZEB1 can also interact with the transcriptional coactivator p300/CBP-associated factor (PCAF), which switches it from a transcriptional repressor to a transcriptional activator, promoting Smad signaling [47]. Additionally, ZEB1 can recruit the Lys-specific demethylase 1 (LSD1), possibly linking it to histone demethylation in EMT [48]. ZEB1 is directly regulated by SNAIL1. Moreover, TWIST1 cooperates with SNAIL1 in the induction of ZEB1 expression [49]. ZEB expression can be induced by TGF-β, WNT and MAPK signaling pathways.

In the past decade, an increasing number of studies have provided strong evidence for the critical role EMT plays in cancer progression and metastasis [50]. EMT allows tumor cells to adapt the constant changes of microenvironment in human tumors and successfully metastasize [6]. Theiry *et al.* proposed a metastasis model in which EMT is activated in primary epithelial tumor cells and allows tumor cells to spread through the body. Then, the tumors undergo mesenchymal to epithelial transition (MET) and form epithelial metastases through increasing epithelial markers including E-cadherin [51].

## 2. Effects of Cigarette Smoke on Cancer Development and Treatment Response

In 1950s, cigarette smoking was first found to be associated with lung cancer as people who smoked around 20 cigarettes a day had 26 times the lung cancer risk than non-smokers, and those who smoked three cigarettes a day had six times greater risk [52]. Since then, the association between cigarette smoke and lung cancer has been studied for more than six decades. Although cigarette smoke contains carcinogens, which convincingly cause lung tumor [53], the adverse effects of smoking are not limited to the lung cancer initiation in smokers. The effect of cigarette smoke on tumor growth and metastasis has been considered. Previous clinical studies reported the relationship between smoking history and cancer spread in lung cancer patients. Continued smoking was found to be associated with a significantly increased risk of mortality and development of a second primary tumor in early stage non-small cell lung cancer (NSCLC) [54]. Cancer patients who were previous or current smokers had an increased risk of metastatic disease at diagnosis [55]. Furthermore, the pro-metastatic effect of cigarette smoke is not found only at disease sites traditionally associated with cigarette smoking such as lung cancer but also at other type of cancer. Previous studies suggest a strong association between smoking behavior and pulmonary metastasis from breast [56], esophageal [57], oropharyngeal [58], and prostate [59] cancers.

Emerging data suggests that nicotine has negative effects on clinical outcome of cancer treatments. Smoking can reduce the efficacy of medical treatment and increase complications during and after surgery and adjunctive therapies [60,61]. Preclinical studies also suggest that nicotine may impair the therapeutic effects of chemotherapy and radiotherapy *in vitro* [62,63] and in a mouse model [64]. Furthermore, patients with limited-stage small-cell lung cancer who continue to smoke during chemotherapy and radiotherapy have poorer survival rates compared with those who do not [65].

However, the mechanism by which cigarette smoking confers drug resistance, reduced therapy efficacy and metastasis remains poorly understood. As EMT has been shown to be associated with drug resistance and metastasis, the link between cigarette smoke and EMT might hold the answer to the molecular network underlying adverse effects of cigarette smoke. It has been reported that there are higher levels of vimentin and other mesenchymal markers [66,67] and a decrease in the expression of E-cadherin in smokers compared with normal non-smokers. Several of the key pathways driving EMT are also aberrantly activated in cancer [68]. Previous *in vitro* studies also indicated that treatment with cigarette smoke extract (CSE) could promote EMT in lung cancer cells [69,70]. Importantly, EMT has been also found to be increased in patients with chronic obstructive pulmonary disease (COPD), which is mainly caused by smoking [67]. The coexistence of lung cancer and chronic obstructive pulmonary disease (COPD) is commonly detected in smokers, and the risk of developing lung cancer in smoking patients is significantly increased in the presence of COPD [71]. Although a detailed discussion of COPD is not within the scope of this review, it is important to emphasize that EMT can play an important role in the link between COPD and lung cancer diseases that are both induced by smoking. One important subsequent outcome with active EMT is the development of angiogenesis [68], a common feature of both lung cancer and COPD [72]. Recently, Fantozzi *et al.* showed that EMT could confer efficient tumorigenicity to breast cancer cells by elevated expression of the pro-angiogenic factor VEGF-A and by increased tumor angiogenesis [73]. Therefore, in this review, we will discuss current knowledge about the molecular mechanisms underlying EMT-inducing effects of cigarette smoke. This research area might provide potential therapeutic agents for targeting EMT in both lung cancer and COPD.

## 3. Molecular Mechanisms of Cigarette Smoke-Induced EMT

### 3.1. The EMT-Inducing Effect of Key Components of Cigarette Smoke

**Figure 1:**
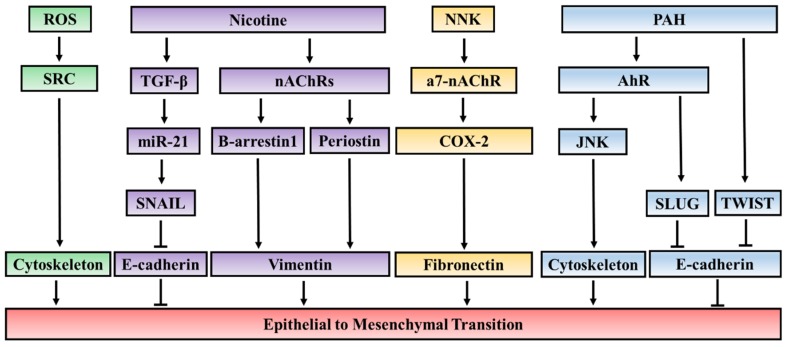
Overview of the molecular networks by which different compounds in cigarette smoke regulate EMT. Reactive oxygen species (ROS) found in cigarette smoke can activate SRC resulting in cytoskeletal modification. Nicotine can induce EMT through nAChRs-dependent and nAChRs-independent pathways. In the nAChRs-dependent pathway, the activation of nAChRs results in the recruitment of β-arrestin1 on to the promoters and promotes the expression of mesenchymal markers. Additionally, the activation of nAChRs also induces the expression of periostin, the overexpression of which has been reported to cause EMT. In the nAChRs-independent pathway, nicotine can induce EMT through upregulating miR-21 in a manner dependent on TGF-β signaling. Nicotine-derived nitrosamine ketone (NNK) has been found to upregulate the expression of the mesenchymal marker fibronectin through cyclooxygenase-2 (COX-2), which is mediated by the activation of a7-nAChR. Polycyclic aromatic hydrocarbons (PAH) can mediate EMT through the activation of aryl hydrocarbon receptor (aryl hydrocarbon receptor), which triggers a marked cytoskeleton remodeling associated with the activation of the JUN *N*-terminal kinase (JNK) pathway. Increased nuclear accumulation of AhR in response to PAH also leads to transcriptional activation of SLUG.

Cigarette smoke contains more than 4000 compounds overall and forty carcinogenic compounds classified into five major classes: Polycyclic aromatic hydrocarbons (PAH), nicotine-derived nitrosamines, aza-arenes, miscellaneous organic compounds, and inorganic compounds [74]. Among these compounds, nicotine, PAH, and nicotine-derived nitrosamine ketone (NNK) have been shown to induce EMT individually in cancer cells through different signaling pathways (Figure 1). The role of other classes of compounds in EMT remains unknown.

#### 3.1.1. Nicotine

Although nicotine is not considered as a carcinogen, recent studies showed that nicotine can contribute to EMT and proliferation in lung cancer through the activation of nicotinic acetylcholine receptors (nAChRs) [75,76] which are expressed in a variety of non-neuronal cells including endothelial cells and several histological types of lung tissue [77]. The binding of nicotine to nAChRs causes the recruitment of β-arrestin and Src to the nicotinic receptors, which activate the MAPK pathway, resulting in induced cell proliferation [49]. Furthermore, nicotine was also found to induce invasion and migration of NSCLC cells through a7-nAChRs [50]. As a hallmark of pro-invasive effects, nicotine could induce changes in gene expression consistent with EMT, characterized by decreased expression of epithelial markers (E-cadherin, ZO-1) and concomitant increase in levels of mesenchymal proteins (vimentin, fibronectin) in both *in vitro* [50] and *in vivo* settings [78] of lung cancer. These results indicated that nicotine, through the nAChR signaling pathway, induces changes in gene expression pattern to facilitate EMT and tumor metastasis. In line with these studies, Pillai *et al.* emphasized the essential role of β-arrestin-1 in nicotine-induced EMT. β-arrestins function as scaffold proteins that recruit a broad spectrum of signaling molecules to membrane-bound receptors [79]. Stimulation of multiple NSCLC cell lines with nicotine led to enhanced recruitment of β-arrestin-1 and E2F1 on promoters and promote the expression of mesenchymal markers [80].

In addition to nAChRs, nicotine was also found to induce EMT through different oncogenic pathways. Nicotine can activate the Wnt3a signal pathway in lung cancer cells, leading to an upregulation of several mesenchymal markers such as vimentin, matrix metalloproteinases-9, and type I collagen as well as downregulation of E-cadherin [81]. This result is consistent with previous studies, which found that Wnt3a activates Wnt/β-catenin signaling and promotes EMT-like phenotypes in breast cancer [82]. Recently, periostin was identified as a novel target involved in nicotine-induced EMT. Periostin, also known as osteoblast-specific factor 2 (OSF-2), is an adhesion molecule that was initially identified in mouse osteoblastic cells as a secreted extracellular matrix protein. Overexpression of the periostin gene has been reported to cause EMT as well as cell migration, invasion and adhesion [83]. Periostin expression was found regulated by nicotine in NSCLC cells through nAChR-independent pathways [84].

#### 3.1.2. Polycyclic Aromatic Hydrocarbons (PAH)

Role of major polycyclic aromatic hydrocarbons (PAH) in EMT induction was first noticed when Benzo[*a*]pyrene, a major PAH, was shown to induce expression of EMT-related genes including plasminogen activator inhibitor-1, fibronectin, TWIST, transforming growth factor-b2, basic fibroblast growth factor in A549 cells [85]. Furthermore, PAH can mediate EMT through the activation of aryl hydrocarbon receptor (AhR) [86]. AhR expression is upregulated in lung adenocarcinoma, suggesting that AhR and its expression might play an important role in the development of lung adenocarcinoma [87]. Prolonged AhR activation triggers a marked cytoskeleton remodeling associated with JNK activation and increased migration [88]. Upon activation by cigarette smoke, increased nuclear accumulation of AhR lead to transcriptional activation of SLUG, resulting in increased EMT.

#### 3.1.3. Reactive Oxygen Species (ROS)

In addition to a majority of carcinogenic and mutagenic chemicals, tobacco smoke also contains stable and unstable free radicals and reactive oxygen species (ROS) in the particulate and the gas phase that can lead to oxidative damages [89]. Although the role of oxidative damage in cancer induced by cigarette smoke is unclear [90], it has been shown that various oxidants present in tobacco smoke such as peroxynitrite [91] and H_2_O_2_ [92] can activate Src, resulting in EMT initiation [93]. Src is the prototypic member of a family of non-receptor membrane associated tyrosine kinases, which are important regulators of a variety of cellular process including cytoskeletal organization, cell–cell contact, and cell–matrix adhesion. Activation of Src family kinases is involved in expression of mesenchymal proteins and other EMT events [94].

### 3.2. Cigarette Smoke Mediated Epigenetic Modifications and Oncogenic Pathways

**Figure 2:**
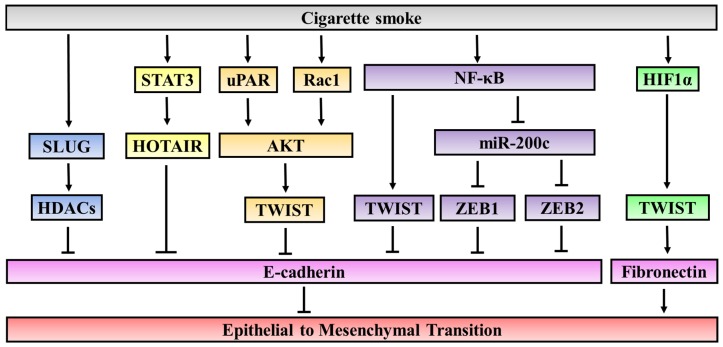
Signaling pathways involved in the inducing effect of cigarette smoke on EMT. Cigarette Smoke Extract (CSE) treatment increases the expression of SLUG, which in turn recruits histone deacetylases (HDACs) to E-cadherin promoter and suppresses E-cadherin expression at the transcriptional level. CSE enhances NF-κB activity, which results in increased expression of TWIST-1 and downregulation of miR-200c. CSE treatment induces secretion of IL-6, leading to the activation of STAT3, which binds to HOTAIR promoter regions and up-regulates HOTAIR. CSE-induced EMT is also found to be accompanied by increased expression of uPAR followed by the activation of AKT. CSE treatment induces hypoxic signaling through the activation of HIF1α, which transcriptionally upregulates the expression of TWIST1. CSE treatment can initiate EMT by increasing the expression of Rac1. Upregulation of Rac1 also leads to an increase in the expression of TGF-β1 and activation of AKT signaling in pulmonary epithelial cells.

In line with the studies on key compounds in cigarette smoke, a number of studies using cigarette smoke extract (CSE) have elucidated various signaling pathways and factors involved in the enhancing effect of cigarette smoke on EMT (Figure 2). Importantly, cigarette smoke can induce EMT in cancer cells through epigenetic modifications or through the activation of oncogenic pathways.

#### 3.2.1. Epigenetic Regulatory Mechanisms

Our previous study showed that exposure of lung cancer cells to CSE induces EMT by downregulating E-cadherin at the transcriptional level. Our results reveal, for the first time, that smoking-mediated decrease in E-cadherin expression plays a key role in the induction in EMT in lung cancer [95]. Smoking-induced EMT is mediated through increased expression of SLUG. Furthermore, the recruitment of HDACs by SLUG is essential for E-cadherin suppression and EMT in cigarette smokers. Treatment with HDAC inhibitor MS-275 reverses CSE-induced EMT, migration, and invasion through the restoration of E-cadherin expression.

Chronic exposure of lung cancer cells to CSE leads to the activation of NF-κB, which is an important signaling pathway in inflammation. Enhancement in NF-κB activity increases the expression of TWIST-1 and downregulates miR-200c, a member of miR-200 family [69]. The miR-200 family (miR-200a, miR-200b, miR-200c, miR-141, and miR-429) has been considered as a novel repressor of EMT [96]. The miR-200 family members function as suppressors of EMT by targeting mRNAs encoding ZEB1 and ZEB2. Expression of the miR-200 family is lost in regions of metaplastic breast cancers lacking E-cadherin, whereas ZEB1 and ZEB2 are highly abundant in invasive mesenchymal cells. This study established a link between inflammatory response and CSE-induced EMT.

Recently, HOTAIR, a long noncoding RNA (lncRNA), was found to be upregulated by CSE and required for CSE-mediated EMT. CSE treatment induces secretion of IL-6, leading to the activation of STAT3, which binds to HOTAIR promoter regions and up-regulates HOTAIR in an autocrine manner [97]. Overexpression of HOTAIR was found in lung cancer tissue and linked to increased metastatic potential [98]. Enforced expression of HOTAIR in epithelial cancer cells induces genome-wide targeting of the Polycomb repressive complex 2, which alters histone H3 lysine 27 methylation, resulting in epigenetic silencing of metastasis suppressor genes [99].

#### 3.2.2. Oncogenic Pathways

CSE-induced EMT was also found to be accompanied by increased expression of uPAR followed by the activation of AKT [100]. uPAR is a glycosylphosphatidylinositol (GPI)-anchored membrane protein that binds urokinase-type plasminogen activator (uPA) [101], which is involved in remodeling of the extracellular matrix, modulating cell adhesion and enhancing cell migration [102]. Increased uPAR expression has been implicated in the promotion of EMT in numerous cancers. uPAR gene silencing was sufficient to block CSE-induced EMT and the activation of AKT signaling [76].

Hypoxia is considered as a poor prognostic factor in non-small cell lung cancer [103]. Chronic exposure to carbon monoxide in cigarette smokers results in tissue hypoxia [104]. Previous studies showed that hypoxia induces an EMT phenotype in lung cancer and increases potential for migration, invasion, and metastasis [105,106]. It has been found that HIF-1 regulates the expression of TWIST by binding directly to the hypoxia-response element (HRE) in the TWIST proximal promoter [107]. CSE treatment induces hypoxic signaling in lung cancer cell lines as well as in mice lung tissue. The activation of HIF1α is important for the upregulation of mesenchymal marker expression and increased production of collagen in response to CSE [108].

CSE treatment can initiate EMT through increasing expression of Rac1 [109]. Rac1 is a member of an important small Rho GTPases family of proteins, which has a major role in cytoskeleton rearrangements and cancer cell metastasis [110]. The activation of Rac1 is critical for TWIST1-induced EMT [111]. Upregulation of Rac1 also leads to an increased TGF-β1 and activation of AKT signaling in pulmonary epithelial cells. Pharmacological inhibition or knockdown of Rac1 decreases CSE exposure induced TGF-β1 release and AKT activation, restraining CSE-induced changes in EMT-related markers [87].

## 4. Conclusion and Perspectives

The abnormal induction of EMT in cancer cells results in a variety of unfavorable biological outcomes including resistance to chemotherapy, enhanced stem cell properties, migration, invasion, and metastasis. In the past decade, it has been well accepted that EMT in normal and tumor epithelial cells has been controlled by complex transcription networks and various post-transcriptional modulators. In this review, we focus on the recent advances in understanding the role of cigarette smoke in EMT induction with emphasis on the following points.

(1) In addition to carcinogenic effects, various compounds found in cigarette smoke such as PAH, nicotine, and ROS can induce EMT through different signaling pathways. The effects can be mediated through specific receptors such as nAChR and AhR for nicotine and PAH, respectively or through other molecular factors. Despite the diversity in signaling pathways and molecules involved in cigarette smoke-induced EMT, the effects are mainly associated with upregulation and enhanced activation of EMT-inducing transcription factors. As these regulators are also found to be associated with poor prognosis in lung cancer, it is essential to get further insight into the molecular network regulated by EMT-inducing cigarette smoke components.
(2) The induction in EMT in cancer by cigarette smoke can be mediated by both changes in epigenetic regulation and activities of a variety of oncogenic signaling pathways. Importantly, several modifications are also linked to biological responses to cigarette smoke, such as inflammation, hypoxia and oxidative stress. Therefore, to effectively study the complex network underlying the effects of cigarette smoke on EMT, it is more applicable to use cigarette smoke extracts to mimic the environmental stresses caused by long-term cigarette smoking. Furthermore, by using CSE, scientists can study the crosstalk between different signaling pathways activated by concerted effects of individual cigarette components in picogram concentrations.
(3) The effect of cigarette smoke on EMT has not only been found in lung cancer, the cancer type directly related to smoking, but also in other cancer types. This is consistent with previous observations that smoking also has adverse effects on health outcomes in patients with different types of cancer. However, it is not well established how cigarette smoking affects cancers other than lung cancer *in vivo*.

The potential of using inhibitors targeting cigarette smoke-induced EMT can be considered. Our previous study showed that using HDAC inhibitor MS-275 can reverse cigarette smoke-induced EMT, migration, and invasion through the restoration of E-cadherin expression [71]. Recently, Epigallocatechin-3-gallate (EGCG), the most abundant polyphenol found in green tea extract, has been reported to have negative effects on migration and epithelial to mesenchymal transition induced by nicotine in NSCLC cells. EGCG reverses the upregulation of HIF1α, vascular endothelial growth factor (VEGF), COX-2, p-AKT, p-ERK and vimentin protein levels, and the downregulation of p53 and beta-catenin protein levels mediated by nicotine in A549 cells. Additionally, a recent study also identified the extracellular-signal-regulated kinase 5 (ERK5) as a negative regulator for CSE-induced EMT [112]. ERK5 is a member of the mitogen-activated protein kinase (MAPK) family and has the Thr–Glu–Tyr (TEY) activation motif [113]. ERK5 is activated by growth factors and has an important role in the regulation of cell proliferation and cell differentiation. Several lines of evidence have suggested that activation of ERK5 suppresses EMT in cancer [114,115].

The persistent smoking after cancer diagnosis has been known to reduce the efficacy of medical treatment and increases complications. However, there are approximately 30 percent of cancer patients who smoked prior to their diagnosis continue to smoke [116]. Therefore, in parallel to placing more efforts for smoking cessation in cancer patients, it is essential to understand molecular mechanisms of the induction of EMT by cigarette smoke and identify novel targets for developing EMT-targeting strategies especially for smoking patients. Altogether, the observations discussed in this review describe a complicated network underlying cigarette smoke-induced EMT. The links between smoking and EMT, including the involvement of oncogenic pathways and epigenetic regulators, are important areas for future study.

## Figures and Tables

**Table 1:** Factors/Proteins involved in epithelial to mesenchymal transition (EMT) and their relevance to lung cancer.

Factors	Description	Relevance to Lung Cancer	Refs
*Transcription factors*			
SNAIL1	Zinc-finger protein, E-box transcriptional repressor	Positive expression associated with poor survival in squamous cell and adenocarcinomas	[8,9,10]
SLUG	Zinc-finger protein, E-box transcriptional repressor	Upregulation associated with poor survival in squamous cell carcinoma	[11]
TWIST1	bHLH factor	Overexpression in primary NSCLCs associated with a shorter overall survival	[12,13,14]
ZEB1	Zinc-finger protein, E-box transcriptional repressor	Higher expression found in metastatic lung tumors compared to primary tumors	[15,16]
FOXC2	Forkhead box transcription factor	Overexpression associated with a worse overall survival and correlated with a shorter recurrence-free survival in patients with stage-I non-small cell lung cancer	[17]
FOXQ1	Forkhead box transcription factor	Upregulation in NSCLC resulting in poor prognosis	[18]
FOXC1	Forkhead box transcription factor	Upregulation correlated with poor tumor differentiation, tumor-node-metastasis stage, and lymph node metastasis in NSCLC patients	[19]
FOXM1	Forkhead box transcription factor	Overexpression associated with poor prognosis of NSCLC patients and tumor metastasis	[20]
*Factors directly associated with EMT*			
E-cadherin	Adhesion glycoprotein	Reduced E-cadherin expression significantly correlated with lymph node metastasis	[21]
N-cadherin	Adhesion glycoprotein	Overexpression associated with a shorter overall survival	[22]
Syndecan-1	Transmembrane (type I) heparan sulfate proteoglycan	High pretreatment serum syndecan-1 level associated with poor prognosis in SCLC treated with platinum-based chemotherapy	[23]
miR-21	Non-coding RNA	Upregulation of serum miR-21 strongly associated with lymph node metastasis and advanced clinical stage of NSCLC	[24]
α-SMA	α-Smooth muscle actin	Overexpression associated with a poor prognosis in patients with clinical stage I-IIIA NSCLC after curative resection	[27]
Vimentin	Member of the intermediate filament family	Upregulation correlated with lymph node metastasis in squamous cell lung carcinoma	[25]
Periostin	Osteoblast Specific Factor	Overexpression associated with decreased progression-free survival	[26]
